# Totally homogeneous networks

**DOI:** 10.1093/nsr/nwz050

**Published:** 2019-04-09

**Authors:** Dinghua Shi, Linyuan Lü, Guanrong Chen

**Affiliations:** 1 Department of Mathematics, College of Science, Shanghai University, Shanghai 200444, China; 2 Institute of Fundamental and Frontier Sciences, University of Electronic Science and Technology of China, Chengdu 610054, China; 3 Department of Electronic Engineering, City University of Hong Kong, Hong Kong, China

**Keywords:** boundary operator, clique vector space, cycle, homology group, totally homogenous network

## Abstract

In network science, the non-homogeneity of node degrees has been a concerning issue for study. Yet, with today's modern web technologies, the traditional social communication topologies have evolved from node-central structures into online cycle-based communities, urgently requiring new network theories and tools. Switching the focus from node degrees to network cycles could reveal many interesting properties from the perspective of totally homogenous networks or sub-networks in a complex network, especially basic simplexes (cliques) such as links and triangles. Clearly, compared with node degrees, it is much more challenging to deal with network cycles. For studying the latter, a new clique vector-space framework is introduced in this paper, where the vector space with a basis consisting of links has a dimension equal to the number of links, that with a basis consisting of triangles has the dimension equal to the number of triangles and so on. These two vector spaces are related through a boundary operator, for example mapping the boundary of a triangle in one space to the sum of three links in the other space. Under the new framework, some important concepts and methodologies from algebraic topology, such as characteristic number, homology group and Betti number, will play a part in network science leading to foreseeable new research directions. As immediate applications, the paper illustrates some important characteristics affecting the collective behaviors of complex networks, some new cycle-dependent importance indexes of nodes and implications for network synchronization and brain-network analysis.

## INTRODUCTION

Network science has gained popularity due to its great achievements over the past 20 years, where small-world networks [1] are built from nearest-neighbor regular networks through rewiring, presenting two significant characteristics of short average path length and large clustering coefficient, while scale-free networks [2] are modeled based on random networks [3], possessing a power-law node-degree distribution. The three fundamental concepts in network science—average path length, degree distribution and clustering coefficient—correspond to three basic structures: chain, star and cycle. The clustering coefficient is calculated based on triangles, but the cycle depends on many more, so there should be a large class of complex networks that have prominent cyclic structures that go beyond the typical small-world and scale-free models. Indeed, one was recently discovered [4], referred to as *totally homogeneous networks*, which was called the ‘$( {k,\ g,\ l})$-homogeneous network’ [4], where *k* is the node-degree variable, *g* is the girth variable (length of the smallest cycle of a node) and *l* is the path-sum variable (sum of all path lengths of a node to other nodes). This new class of networks were coined as the type of networks that have optimal synchronizability if it has a maximum *g* and a minimum *l*, which is a particularly important type of regular network that optimizes the network synchronizability [4], as well as controllability and robustness against attacks. Despite their usefulness in various applications (as further discussed below), it is technically challenging to find such networks, since they require all nodes to have the same degree, the same girth and the same path-sum. Yet small-sized totally homogeneous sub-networks ubiquitously exist in all complex networks, typically triangles and smallest *k*-cavities (to be further discussed below). Notably, totally homogeneous networks have natural symmetries, signifying their importance in network theory and applications, and therefore should be more carefully investigated.

**Figure 1:**
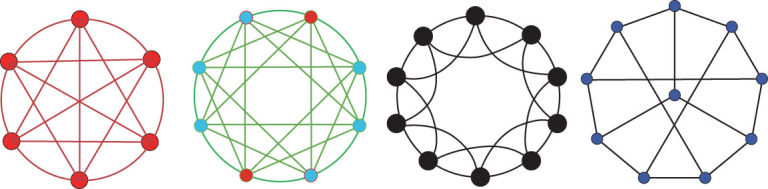
A 6-node fully connected network; an 8-node smallest 3-cavity network; a 10-node nearest-neighbor regular network; a 10-node sync-optimal network.

A network usually has fully connected sub-networks (i.e. complete sub-graphs) like triangles and tetrahedrons, called simplexes in topology or cliques in graph theory, which are special totally homogeneous (sub)networks. Here, a node is a 0-clique, a link is a 1-clique, a triangle is a 2-clique, a tetrahedron is a 3-clique and so on. Other than cliques and nearest-neighbor regular (sub)networks, it is important to find those non-fully connected and yet linearly independent cavities as well as other sync-optimal totally homogeneous (sub)networks, which are known to be very important in brain functional networks and big-data analysis, as will be further discussed below. Typical examples of totally homogeneous (sub)networks, namely a fully connected network, a smallest *k*-cavity network, a nearest-neighbor regular network and a sync-optimal network, are shown in Fig. 1.

A network can be represented by an adjacency matrix, in which element 1 means two corresponding nodes are connected by a link, while 0 means not. Therefore, the node degree is of the highest importance for a network and, as a result, the emergence of giant nodes (hubs) signifies the key role of stars in scale-free networks, which has been a main research subject in the past.

By examining cycles, it is easy to find that cliques, such as links and triangles, constitute the backbone of a network. Therefore, a new clique vector-space framework in the form of a sequence of vector spaces defined on the binary field is introduced. Let ${C_0}$ be the vector space with a basis consisting of all nodes, ${C_1}$ be one with a basis of all links (edges), ${C_2}$ be the one with a basis of all triangles and so forth. Noting that the three links of a triangle in ${C_2}$ are links in ${C_1}$, a boundary operator is defined between these two vector spaces, so that the two vector spaces connected by the boundary operator can be described and analysed using a boundary matrix. It will be seen that boundary matrices have richer mathematical contents and are useful tools for advanced studies. For instance, one can introduce the concept of linear dependence for chain vectors and for cycle vectors, as well as equivalence between cycles, so that the resulting chain groups and cycle groups (including boundary groups and homology groups) can be investigated by using advanced mathematical tools from algebraic topology and group theory. It can be foreseen that such a clique vector-space framework will enrich the studies of network science and meanwhile bring up more opportunities to the field.

Using tools from algebraic topology can help find invariants in networks. The most well known is perhaps the Euler characteristic number, which equals the alternating sum of all simplexes in the network, namely the number of nodes minus the number of links then plus the number of triangles and then minus the number of tetrahedrons … until there are no more to add or subtract [5]. Another important invariant is the Betti number, which is the total number of linearly independent cavities [5], defined as follows: the order-0 Betti number is the number of connected sub-networks, order-1 Betti number is the number of linearly independent 1-cavities (which are non-triangular cycles) and so on, as will be further illustrated by examples below. The Euler-Poincaré formula connects these two indexes together [5]: the alternating sum of simplexes equals the alternating sum of the Betti numbers. Besides, the number of links in a spanning tree of a connected network equals the number of all nodes minus 1, which is the rank of the chain group in the network [6]. Moreover, the rank of the cycle group, namely the number of linearly independent triangular and non-triangular cycles, equals the number of links minus the number of nodes and then plus 1 [6].

Cycles have been a main research subject in graph theory [6], while algebraic topology is an important branch in mathematics [5]. In the current literature of network science, however, investigations involving both network cycles and algebraic topology are quite rare. Nevertheless, there is some significant progress: the finding of a criterion for network synchronizability (2002) [7]; the introduction of (sync-optimal) totally homogeneous networks (2013) [4]; the study of signals and noise in brain activities (2017) [8]; the discovery of cliques and cavities in brain functional networks (2018) [9]; the search for basic cycles in measuring the importance of a node and cycle index in spreading over WeChat and their relationships with hyper-networks (2019) [10].

This paper presents the mathematical description of a new framework of clique vector spaces, first introducing related concepts of various cycles, second defining a sequence of clique vector spaces associated with boundary operators and finally discussing the chain group, cycle group, boundary group and homology group. Then, it shows how to utilize boundary matrices to calculate linearly independent cycles and cavities of different orders, as well as their characteristic indexes, in a complex network. Furthermore, by examining the key factors that affect the collective behaviors of a network, it demonstrates the important role of totally homogeneous sub-networks in a complex network. Finally, it briefly discusses some applications of algebraic topology in network science, indicating a couple of future research topics.

## RESULTS AND DISCUSSION

### Description of the new mathematical framework

Investigating network cycles is much more difficult than examining node degrees, since cycles have many variants such as higher-order cycles, linearly (in)dependent cycles and redundant cycles that contain smaller circles. A simple cycle is intuitively clear, which is a closed path starting from a node and returning to the same node after traversing some other nodes. The smallest cycle is the triangle, which is also a clique, called 2-clique, and a tetrahedron is 3-clique. The 3-clique is not a cycle in the usual sense, and is called a second-order cycle, or simply a 2-cycle. Similarly, 4-clique is a 3-cycle, 5-clique is a 4-cycle and so on. Yet, 0-clique and 1-clique are not cycles. The concept of linearly (in)dependent cycles or cavities will be introduced through the description of the new framework next.

The new framework of clique vector spaces in the binary field and their associated boundary operators are defined as follows. Let ${C_k}$ be the vector space with a basis consisting of *k*-cliques, with dimension ${m_k}$ equal to the number of the *k*-cliques. The Euler characteristic number $\chi \ = {m_0}{\rm{\ }} - {m_1} + {m_2} - \cdots$. All the vectors in ${C_k}$ are subsets of some *k*-cliques, with the empty set being the zero vector, denoted as $\emptyset$ or 0. In the binary field, there are only two elements, 0 and 1, with $1 + 1\ = \ 0.$ The addition between two vectors *c* and *d* is defined by set operations as $c + d\ = ( {c \cup d} )\ - ( {c \cap d} )$.

Define a boundary operator ${\partial _k}\!\!:\ {C_k} \to{C_{k - 1}}$ to connect two successive vector spaces in the following way: denote triangle $( {1,2,3} ) \in{C_2}$ by ${\sigma _{123}} \in{C_2}$, which has boundaries $( {1,2} ),\ ( {2,3} ),\ ( {3,1} )$ and define ${\partial _2}\ ( {{\sigma _{123}}} ) = {\sigma _{12}}\ + {\sigma _{23}} + {\sigma _{31}}$, where the ‘+’ operation is performed in the binary field. For the two end nodes of the boundary $( {1,2} )$, node 1 and node 2, one has ${\partial _1}\ ( {{\sigma _{12}}} ) = {\sigma _1}\ + {\sigma _2}$. Again, by using additions in the binary field, one obtains

$$\begin{eqnarray*}{\partial _1}\ \left( {{\partial _2}\left( {{\sigma _{123}}} \right)} \right) = {\partial _1}\ \left( {{\sigma _{12}} + {\sigma _{23}} + {\sigma _{31}}} \right) = {\sigma _1}\\ + {\sigma _2} + {\sigma _2} + {\sigma _3} + {\sigma _3} + \ {\sigma _1} = \ 0.\end{eqnarray*}$$

**Figure 2:**
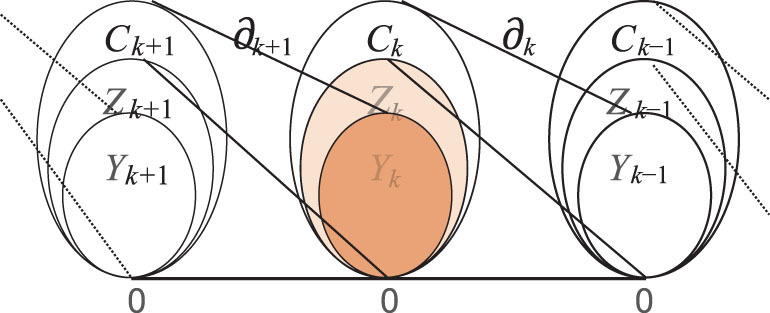
Relationships of a sequence of vector spaces in a network, with boundary operators and associated subspaces (See [5]).

Since ${C_1}$ is a vector space consisting of links, its elements are called 1-chains. If, for $l \in{C_1}$, the chain satisfies ${\partial _1}\ ( l ) = \ 0,$ then it is called a 1-cycle. Thus, a *k*-cycle *l* is defined by ${\partial _k}\ ( l ) = \ 0$. Further, define the kernel of ${C_k}$ by $\ker ( {{\partial _k}} ) = {\rm{\{ }}l \in{C_k}\ {\rm{|}}\ {\partial _k}\ ( l ) = \ 0\}$, denoted as ${Z_k}$, which is called the null space or kernel space. Moreover, ${\rm{im\ }}( {{\partial _{k + 1}}} ) = {\rm{\{ }}{\partial _{k + 1}}( l )\ {\rm{|}}\ l \in{C_{k + 1}}\}$ is the image of ${C_{k + 1}}$ mapping to ${C_k}$, denoted as ${Y_k}$, called the image space on ${C_k}$. Clearly, both ${Z_k}$ and ${Y_k}$ are subspaces of ${C_k}$. Since ${\partial _k}\ ( {{\partial _{k + 1}}} ) = \ 0$, one has ${\rm{im}}( {{\partial _{k + 1}}} ) \subseteq{\rm{ker}}( {{\partial _k}} )$. The containment relationships of these subspaces are shown in Fig. 2.

To study the linear dependence, boundary matrices are introduced. For instance, in ${C_1}$, one can define a node-link matrix ${B_1}$, in which an element is 1 if the node is in the link correspondingly; otherwise, it is 0. Similarly, define a link-face matrix ${B_2}$ on ${C_2}$, in which an element is 1 if the link is on the face correspondingly; otherwise, it is 0. Through elementary row (column) transformations in the binary field, one can calculate the rank ${r_k}$ of the boundary matrix ${B_k}$, which is the number of linearly independent vectors in ${C_k}$. Moreover, all *k*-chains in ${C_k}$ constitute an Abel group, called a chain group, with the empty set being the zero element. The number of generating elements of a chain group is the rank ${r_k}$ of the boundary matrix ${B_k}$. Likewise, all the *k*-cycles of ${\rm{ker}}( {{\partial _k}})$ and all *k*-boundaries of ${\rm{im}}( {{\partial _{k + 1}}} )$ each form an Abel group, called cycle group ${Z_k}$ and boundary group ${Y_k}$, with ranks ${m_k} - {r_k}$ and ${r_{k + 1}}$, respectively. Two *k*-cycles *c* and *d* are said to be equivalent, denoted as $c\sim d$, if $c + d$ is a boundary of a $( {k + 1} )$-chain. All equivalent cycles constitute an equivalent class. By definition, if $b \in{Y_k}$, then $b\sim\emptyset$. Decomposing the cycle group ${Z_k}$ via the boundary group ${Y_k}$ yields a homology group ${Z_k}/{Y_k}$, with rank equal to the Betti number ${\beta _k} = {m_k}\ - {r_k} - {r_{k + 1}}$. Elements of this homology group are cycle-equivalent classes, called *k*-cavities.

Next, the above-introduced concepts and algorithm are illustrated by a simple network. See Supplementary Data, Section 1 for detailed computations.

**Figure 3:**
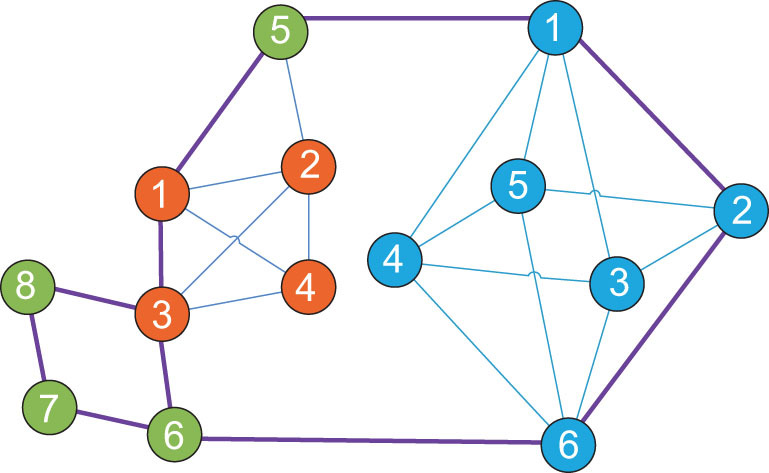
A network with 14 nodes and 26 links.

The network in Fig. 3 has 13 triangles and 1 tetrahedron, so its Euler characteristic number is $\chi \ = \ 14 - 26 + 13 - 1\ = \ 1 - 2 + 1\ = \ 0$, where the Betti numbers are calculated in Supplementary Data, Section 1. Since ${\beta _1} = \ 2$, the homology group ${Z_1}/{Y_1}$ has rank 2 and order 4, with four cycle-equivalent classes: empty set $\emptyset$ (including 13 triangles), two 1-cavities and their sum. The key is to search for these cliques and cavities. For distinction below, the right-side node numbers are marked by apostrophes.

#### Finding a spanning tree of the network

Links (1,2) (1,3) (1,4) (1,5) (3,6) (3,8) (6,7), (5,1’), (1’,2’) (1’,3’) (1’,4’) (1’,5’) (2’,6’) constitute a spanning tree, where a number with prime also indicates a node, which is not unique.

#### Searching for linearly independent cycles of the network

Joining other links to the spanning tree forms linearly independent cycles, with the total number equal to the number of links minus the number of nodes and then plus 1. In so doing, linearly dependent cliques and equivalent cavities with the same length in the network should also be included.

Joining links (2,3) (2,4) (2,5) yields (1,2,3) (1,2,4) (1,2,5), respectively.
Joining link (3,4) yields (1,3,4) and backtracking to (2,3,4)**(1,2,3,4)** (3-clique).
Joining link (7,8) yields (3,6,7,8).
Joining link (6,6’) yields (1,5,1’,2’,6’,6,3), and backtracking to (2,5,1’,2’,6’,6,3).
Joining links (2’,3’) (3’,4’) (4’,5’) (5’,2’) yields (1’,2’,3’) (1’,3’,4’) (1’,4’,5’) (1’,5’,2’).
Joining link (3’,6’) yields (2’,3’,6’), and backtracking to (1,5,1’,3’,6’,6,3) (2,5,1’,3’,6’,6,3).
Joining link (4’,6’) yields (3’,4’,6’), and backtracking to (1,5,1’,4’,6’,6,3) (2,5,1’,4’,6’,6,3).
Joining link (5’,6’) yields (4’,5’,6’), and backtracking to (5’,2’,6’) (1,5,1’,5’,6’,6,3) (2,5,1’,5’,6’,6,3)**(1’,2’,3’,4’,5’,6’)** (2-cavity).

Here, those cycles without underlines are linearly independent 1-cycles. Obviously, all 1-cycles and 2-cycles are totally homogeneous sub-networks.

To this end, it should be clear that the above analytic and computational methods can be extended to directed and weighted complex networks, even multi-layered networks.

Next, the invariants and linearly independent cycles of the four totally homogeneous networks shown in Fig. 1 are computed:

(1) The 6-node fully connected network (5-clique)

Characteristic number: $\chi\! = \mathop \sum _{i = 0}^5 {( { - 1} )^i}C_6^{i + 1} =\! 1$
Betti numbers: ${\beta _0} = {\rm{\ }}1$, ${\beta _1} = {\rm{\ }} \cdots{\rm{\ }} = {\beta _5}{\rm{\ }} = {\rm{\ }}0$
Linearly independent cycles: $15 - 6 + 1{\rm{\ }} = {\rm{\ }}10$

(2) The 8-node smallest 3-cavity is a closed structure enclosed by tetrahedrons

Characteristic number: $\chi = 8 - 24 + 24 - 8 = 0$
Betti numbers: ${\beta _0} = \ 1$, ${\beta _1} = {\beta _2}\ = \ 0$, ${\beta _3} = \ 1$
Linearly independent cycles: $24 - 8 + 1\ = \ 17$

(3) The 10-node nearest-neighbor regular network

Characteristic number: $\chi \ = \ 10 - 20 + 10\ = \ 0$
Betti numbers: ${\beta _0} = \ 1$, ${\beta _1} = \ 1$, ${\beta _2} = \ 0$
Linearly independent cycles: $20 - 10 + 1\ = \ 11$

(4) The 10-node sync-optimal network

Characteristic number: $\chi \ = \ 10 - 15 + 0\ = \ - 5$
Betti numbers: ${\beta _0} = \ 1$, ${\beta _1} = \ 6$, ${\beta _2} = \ 0$
Linearly independent cycles: $15 - 10 + 1\ = \ 6$

The four representative totally homogeneous networks shown in Fig. 1 have characteristic numbers as follows:

Characteristic number of a simplex: $\chi \ = \ 1$
Characteristic number of a smallest *k*-cavity:
  $$\begin{eqnarray*}\chi &=& 1 + {( { - 1} )^k}\end{eqnarray*}$$
Characteristic number of a regular network: $\chi \ = \ 0$
Characteristic number of a sync-optimal network ofdegree 3:
  $$\begin{eqnarray*}\chi &=& - n/2,n = 6,8,10,\ \ldots\end{eqnarray*}$$

It is observed that the characteristic number of a smallest *k*-cavity oscillates between 0 and 2, depending on whether the *k* is odd or even, perhaps due to the various dynamic properties of such networks (‘topological cavities of different dimensions, around which information may flow in either diverging or converging patterns’ [9]). It is also noted that the characteristic number of a sync-optimal network is always negative.

### Main factors affecting collective behaviors of a complex dynamical network

The seminal paper [1] on small-world networks studies the collective behaviors of a dynamical network. It points out that regular networks have relatively large clustering coefficients, but their average path lengths are generally quite long, and that random networks are opposite. So, it concludes that both are not good for collective dynamical behaviors such as information spreading and multi-agent synchronization. Thereby, it recommends a small-world network model that has both advantages of large clustering coefficients and short average path lengths.

Now, focusing on cyclic structures in small-world networks reveals some interesting phenomena that have not been observed or emphasized before.

#### Network synchronization—characteristic number is key

**Figure 4:**
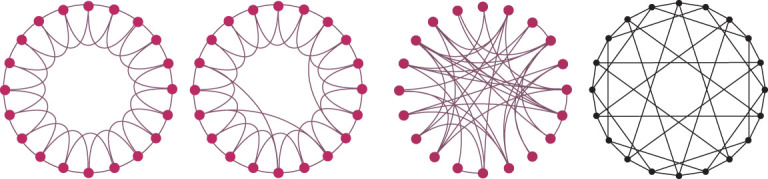
Nearest-neighbor regular network, small-world network, random network and sync-optimal network.

In the study of optimal synchronizability of complex networks, it was found [4] that the totally homogeneous network with equal node degree, long girth and short path-sum is the best. Here, the synchronizabilities of four typical networks shown in Fig. 4 are compared: regular network, small-world network, random network and sync-optimal totally homogeneous network, in which the small-world network is created through random rewiring [1] while the sync-optimal network is created through deterministic rewiring [4]. All these sample networks are connected with 20 nodes and 40 links without tetrahedrons.

Data about these four sample networks are summarized below:

(1) Nearest-neighbor regular network

Triangles: 20
Characteristic number: $\chi \ = \ 20 - 40 + 20\ = \ 0$
Betti number: ${\beta _0} = \ 1$, ${\beta _1} = \ 1$, ${\beta _2} = \ 0$

(2) Small-world network

Triangles: 17
Characteristic number: $\chi \ = \ 20 - 40 + 17\ = \ - 3$
Betti number: ${\beta _0} = \ 1,$${\beta _1} = \ 4$, ${\beta _2} = \ 0$

(3) Random network

Triangles: 15
Characteristic number: $\chi \ = \ 20 - 40 + 15\ = \ - 5$
Betti number: ${\beta _0} = \ 1$, ${\beta _1} = \ 6$, ${\beta _2} = \ 0$

(4) Sync-optimal network

Triangles: 0
Characteristic number: $\chi \ = 20 - 40 + 0 = - 20$
Betti number: ${\beta _0} = \ 1$, ${\beta _1} = 21$, ${\beta _2} = \ 0$

To compare their synchronizabilities, the eigenvalues of their Laplacian matrices are calculated [4,7]: the nearest-neighbor regular network has spectral gap (smallest nonzero eigenvalue) 0.4799 with eigen-ratio (smallest nonzero eigenvalue versus largest eigenvalue) 0.0769; the small-world network has spectral gap 0.5035 and eigen-ratio 0.0714; the random network has spectral gap 0.7947 and eigen-ratio 0.0812; the sync-optimal network has spectral gap 2.0000 and eigen-ratio 0.2982.

These results depict that the network synchronizabilities of the regular network, small-world network, random network and sync-optimal network are increasing successively. In particular, it shows that the key factor affecting the network synchronizability should be the Euler characteristic number: the smaller the characteristic number, the better the synchronizability for networks of the same size. Furthermore, the characteristic number depends on both 2-cliques and 1-cavities: having fewer 2-cliques but more 1-cavities, the characteristic number will be smaller, hence the network is easier to synchronize. These data are consistent with the previous observations. Note also that the clustering coefficient depends on the number of triangles, so a larger clustering coefficient means more 2-cliques are involved, and consequently the network synchronizability will become worse. Relatively to a larger clustering coefficient, a shorter average path length is more important for better network synchronization.

#### Network spreading—totally homogenous networks are better

Now, consider information or disease spreading on the four networks shown in Fig. 4, all based on the cyclic Susceptible-Infected-Recovered (SIR) model [10]. This cycle-based SIR model differs from the conventional SIR model [11] in that the nodes belonging to the same cycle can always transmit information or disease, even if they are not directly connected to each other.

The performed simulations involved successively selecting every node as the source and, with spreading probability 0.06 and recovering probability 1.00, processing the information though nodes with S (susceptive), I (infected) and R (recover) states until no more infected nodes remained in the network. Then, after 100 runs, the average number of recovered nodes was recorded. The final results were Network 1: 2.656 nodes, Network 2: 2.441 nodes, Network 3: 1.349 nodes and Network 4: 4.111 nodes (see Supplementary Data, Section 2 for more details).

These simulation results clearly show that the random network is the worst, the small-world network is not as good as the regular network and the totally homogeneous network is the best.

### Other promising applications based on cycles

#### Cycle-based importance indexes of nodes—cycle number and cycle ratio

There are many indexes for measuring the importance of a node in a network [12], but there do not seem to be any based on cycle structure.

Two new concepts of cycle number and cycle ratio have been recently introduced [10] for measuring the importance of nodes. A node may have several smallest cycles, called the smallest basic cycles of the network. All non-redundant cycles are called basic cycles. The cycle number of a node is defined as the total number of basic cycles that pass this node. The cycle ratio of a node *i* is then defined as the sum of the proportions of this node *i* appearing in the basic cycles of all those nodes that are contained in the basic cycles of this node *i*.

To evaluate the performances of the indexes’ abilities to measure the node importance in a network, three existing indexes (degree, H-index and coreness) and the two new indexes (cycle number and cycle ratio) are compared [10] to study their effects on the connectivity and spreading over a network. For connectivity, all nodes are ranked according to their importance measured by an index and some nodes are removed, then a portion of nodes in a giant surviving sub-network is computed and finally their relationships are plotted for comparison. For nodes with the same index value, randomly rank them one after another. Simulation demonstrates that the intentional attack according to the cycle ratio ranking is more effective. For spreading, choose the initial node according to the importance ranking, from high to low, as the source node. The SIR spreading process is then performed until no infected node remains. After 100 runs, the average number of recovered nodes is recorded and the Kendall's tau correlation coefficient [13] is calculated. In evaluating the spreading performance, due to the cyclic structure, the spreading matrix is used instead of the adjacency matrix. Here, the spreading matrix is introduced from WeChat data, which means that two nodes in the same group can communicate, even if they do not know each other. Results show that infected nodes spread very well according to the cycle-number ranking on the cycle-based SIR model.

#### Relation of network and hyper-network—studying hyper-networks

**Figure 5.**
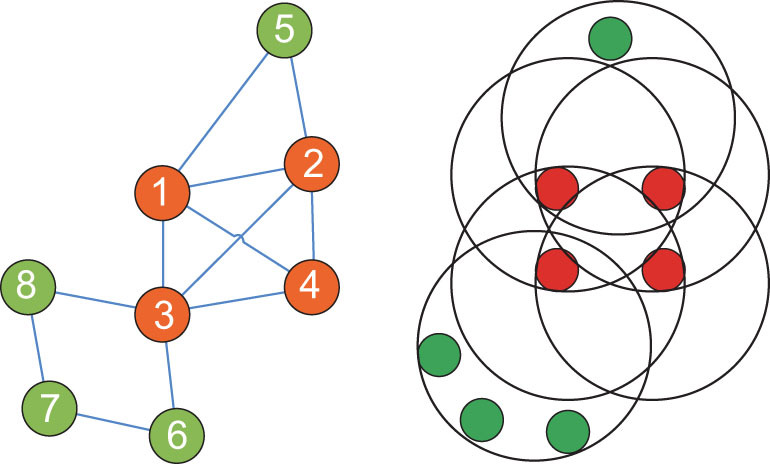
An ordinary network (left) and its converted hyper-network (right).

In classical graph theory, one link can only connect two nodes. In reality, however, one link could be shared by multiple nodes. Such a link is called a hyperlink. A network consisting of nodes and hyperlinks is called a hyper-network. There exists correlation of an ordinary network and a hyper-network [10]. By viewing a basic cycle of a node as a hyperlink, an ordinary network can be converted into a hyper-network, as shown in Fig. 5. The questions are whether the reverse can be performed and, if so, whether the reverse process preserves all information. Since ordinary networks are special cases of hyper-networks, it is clear that generally the answers are no. But, this does not exclude particular situations. In fact, for a hyper-network, multiplying the incidence matrix by its transpose yields a cycle-number matrix. Then, dividing each row of the cycle-number matrix by the cycle number yields a cycle-ratio matrix, and then adding each column to it gives the cycle ratio of each node. Thus, the new notion sheds some light on future research on hyper-networks.

#### Brain functional networks—important roles of cliques and cavities

It was pointed out [9] that, although the human brain looks sparsely connected, its clique structure therein is quite dense. It was found [9] that cliques play a very important role in cortical, visual and perceptive functions. However, from the conventional graph-theoretical viewpoint, one can only observe the connectivity among nodes and can not discover deeper and higher-order structural characteristics of the brain, which needs more powerful mathematical tools such as algebraic topology. From an empirical research investigation, it was found [9] that cycles with longer girths are extremely important in the task of controlling the brain and that the cavity structure is even more critical in the spreading patterns of the brain. Surprisingly, it was found [9] that the universal cavity structure in the brain does not exist in the conventional benchmark null network model, indicating the need for more powerful topological graph theory [14] beyond the classical algebraic graph theory in the studies of the brain.

#### Computational topology coming to network science—looking for higher-order topological features

Persistent homology [15], an important subject in algebraic topology, can be used to improve computational accuracy in different spaces and to detect subtle details in a multi-scale space, recovering more essential features of a research object on the ground space. In contrast, the conventional techniques such as signal sampling and noise analysis as well as parameter selection may yield some false results. In a study of the functional network formed by time-series data obtained by a weighted rank filtration technique, it was found [8] that cliques and cavities in a functional network have higher-order characteristics than the connectivity among nodes, which provides much more useful information, consistent with some existing studies [9]. By investigating the synchronization of Kuramoto oscillators using fMIR data, it was found [8] that persistent homology can reveal clearly some synchronous behaviors in the learning process of the brain that were not discovered by conventional signal sampling and noise analysis. Typically, persistent homology helps to distinguish strong and weak synchronization phenomena in communities of the brain network and helps to detect functional changes through the learning process of the brain. A recent report [16] shows that persistent homology can be used to assist in topological data analysis, to reveal local, mesoscale and global properties and features of the network, using weighted, noisy and non-uniformly sampled complex data, verified by Electroencephalography (EEG) data analysis.

## CONCLUSION

Using a sequence of clique vector spaces alongside boundary operators to describe complex networks has demonstrated well that cliques, simplexes and fully connected sub-networks are the backbones of various networks. This framework allows higher-level mathematical concepts and methods such as characteristic number, homology group and Betti number to play more significant roles in network-science studies. They provide useful tools for uncovering and analysing higher-order topological features and global structures of a complex network. Four representative classes of totally homogeneous networks have been examined, especially some elegant properties of the smallest *k*-cavity sub-networks and sync-optimal networks, revealing that cycle homogeneity is as important as node heterogeneity for understanding complex networks. Network-synchronization criteria originate from physics and are then evolved via optimization to establish the notion of totally homogenous networks and finally connected to some invariants in algebraic topology, with significance demonstrated by brain research. This process highlights the interactions among network science, physics, biology and mathematics. When looking at an object from different angles, one finds different aspects about it. The situation resembles what the famous Chinese poet Su Dongpo said in his well-known poem [17], ‘From the side, a whole range; from the end, a single peak: far, near, high, low, no two parts alike. Why can’t I tell the true shape of Lu-shan? Because I myself am in the mountain’. The new perspective of this paper hopefully will open up a new research direction in network-science studies in the near future.

## Supplementary Material

nwz050_Supplemental_File
