# Supplementary material for: Totally homogeneous networks
Source: Natl Sci Rev. 2019 Apr 9;6(5):962–9. doi: 10.1093/nsr/nwz050 (PMC8291615; doi:10.1093/nsr/nwz050)
Supplement: nwz050_Supplemental_File [file nwz050_supplemental_file.docx]

**Supplementary Information**

**Totally Homogeneous Networks**

Dinghua Shi^1^, Linyuan Lü^2^, and Guanrong Chen^3,*^

^1^Shanghai University, Shanghai 200444, China

^2^University of Electronic Science and Technology of China, Chengdu 611731, China

^3^City University of Hong Kong, Hong Kong, China

**Section 1**: Illustrative examples.


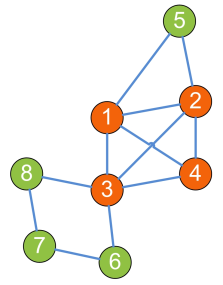

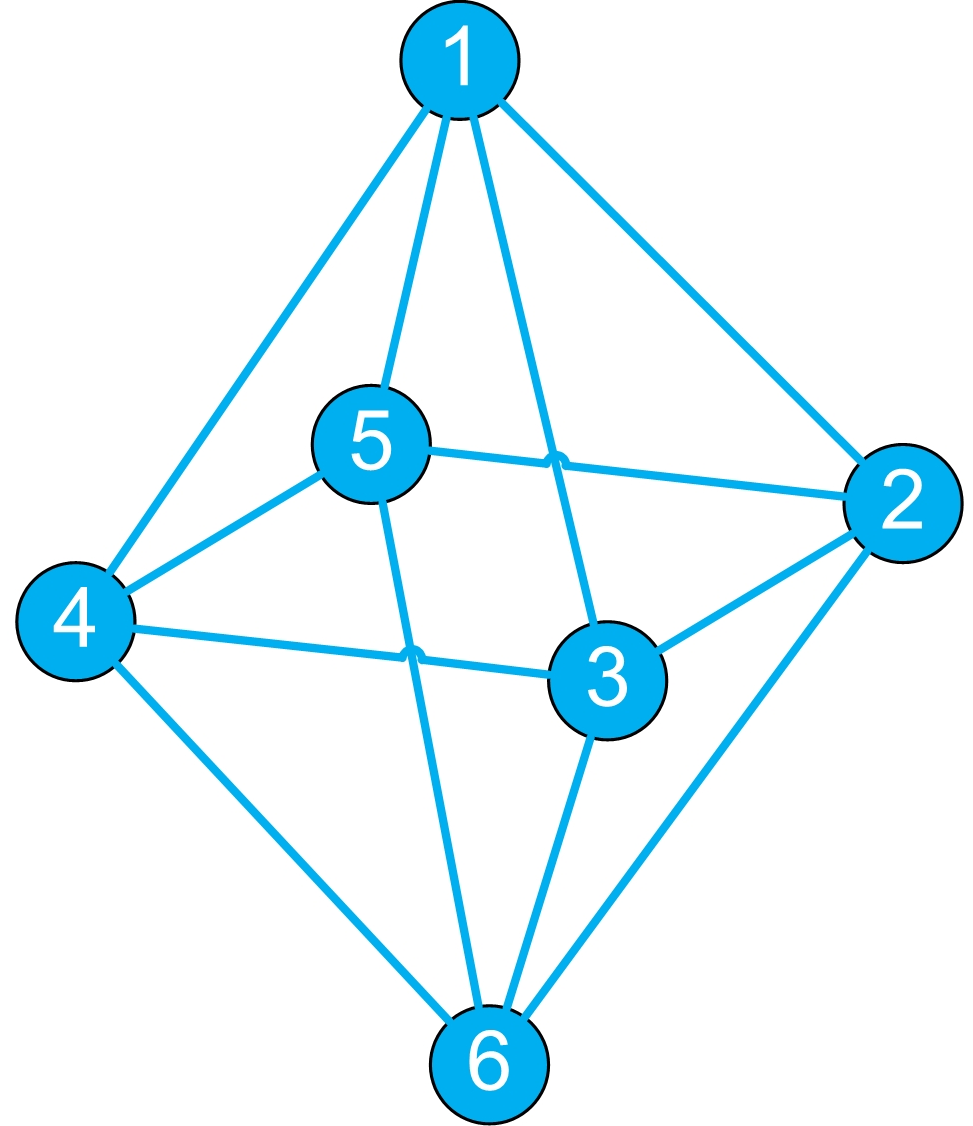


Figure 6. Left: a heterogeneity network; Right: a homogeneity network.

**Example 1.**

Consider the connected undirected network shown on the left side of Fig. 3, which has 8 nodes and 12 links.

A basis of $C_{1}$ is { (1,2)，(1,3)，(1,4)，(1,5)，(2,3)，(2,4)，(2,5)，(3,4)，(3,6)，(3,8)，(6,7)，(7,8) }, and so its dimension is $m_{1}=12$. By definition, the node-link matrix $B_{1}$ is as follows, which has rank $r_{1}=7$ by checking on the binary field.

(1,2) (1,3) (1,4) (1,5) (2,3) (2,4) (2,5) (3,4) (3,6) (3,8) (6,7) (7,8)

1 1 1 1 1 0 0 0 0 0 0 0 0

2 1 0 0 0 1 1 1 0 0 0 0 0

3 0 1 0 0 1 0 0 1 1 1 0 0

4 0 0 1 0 0 1 0 1 0 0 0 0

5 0 0 0 1 0 0 1 0 0 0 0 0

6 0 0 0 0 0 0 0 0 1 0 1 0

7 0 0 0 0 0 0 0 0 0 0 1 1

8 0 0 0 0 0 0 0 0 0 1 0 1

In this matrix, all the red number “1”s together correspond to a spanning tree of the network, consisting of links $\left( 1,2 \right), \left( 1.3 \right), \left( 1,4 \right), \left( 1,5 \right), \left( 3,6 \right), \left( 3,8 \right), (6,7)$, where the number of links is equal to the number of nodes minus 1. Together with the other links corresponding to black number “1”s, they form linearly independent cycles, i.e., cliques $\left( 1,2,3 \right), \left( 1,2,4 \right), \left( 1,2,5 \right), (1,3,4)$ and cavity $(3,6,7,8)$, where the number of cycles equals the number of links minus the number of nodes and then plus 1. In this network, there is a smallest cycle $(2,3,4)$, which is a linearly dependent cycle because

$\left( \sigma_{12}+\sigma_{23}+\sigma_{31} \right)+\left( \sigma_{12}+\sigma_{24}+\sigma_{41} \right)+\left( \sigma_{13}+\sigma_{34}+\sigma_{41} \right)+\left( \sigma_{23}+\sigma_{34}+\sigma_{42} \right)=0$.

**Remark:** The first ellipse $C_{1}$ shown in Fig. 2 is a chain group, where the 12 links can form many 1-chains and 1-cycles, in which there are 7 linearly independent chains, namely $\sigma_{12}, \sigma_{13}, \sigma_{14}, \sigma_{15}, \sigma_{36}, \sigma_{38}, \sigma_{67}$ and they form the first sell with rank $r_{1}=7$. The second ellipse $Z_{1}$ is a cycle group, which has 5 linearly independent 1-cycles, with rank $m_{1}-r_{1}=5$. The third ellipse $Y_{1}$ is a boundary group, consisting of 4 linearly independent cycles, $\sigma_{12}+\sigma_{23}+\sigma_{31}$, $\sigma_{12}+\sigma_{24}+\sigma_{41}$, $\sigma_{13}+\sigma_{34}+\sigma_{41}$, $\sigma_{12}+\sigma_{25}+\sigma_{51}$, with rank $r_{2}=4$, as further illustrated below. There is only one linearly independent cavity in the second sell, $\sigma_{36}+\sigma_{67}+\sigma_{78}+\sigma_{83}$ with rank given by the Betti number $\beta_{1}=m_{1}-r_{1}-r_{2}=1$. This subnetwork has only one connected branch, with Betti number $\beta_{0}=1$. Homological group $Z_{1}/Y_{1}$ has two elements: the empty set and a cavity, with rank $\beta_{1}=1$ and order 2.

Higher-order trees and linearly independent cycles can be similarly obtained. For example, a basis of $C_{2}$ is $\{\left( 1,2,3 \right), \left( 1,2,4 \right), \left( 1,3,4 \right), \left( 2,3,4 \right), \left( 1,2,5 \right)\}$, with dimension $m_{2}=5$. In $C_{2}$, the link-face matrix $B_{2}$ is as follows, with rank $r_{2}=4$.

(1,2,3) (1,2,4) (1,3,4) (2,3,4) (1,2,5)

(1,2) 1 1 0 0 1

(1,3) 1 0 1 0 0

(1,4) 0 1 1 0 0

(1,5) 0 0 0 0 1

(2,3) 1 0 0 1 0

(2,4) 0 1 0 1 0

(2,5) 0 0 0 0 1

(3,4) 0 0 1 1 0

In this matrix, the triangles corresponding to the red “1”s, constitute a spanning tree of the network. Here, the four triangles $\left( 1,2,3 \right), \left( 1,2,4 \right), \left( 1,3,4 \right), (1,2,5)$ constitute a 2-chain, in which the number of triangles is equal to the matrix rank $r_{2}=4$. The three triangles in front, together with the triangle $(2,3,4)$, form a 2-cycle, since $\partial_{2}\left( \sigma_{123}+\sigma_{124}+\sigma_{134}+\sigma_{234} \right)=0$. The 2-circle is a closed structure enclosed by triangles. With the number of cycles equal to$m_{2}-r_{2}=1$, the 3-clique $\sigma_{1234}$ is the only 2-cycle, i.e. $m_{3}=1$, thus $r_{3}=1$. Since the Betti number is $\beta_{2}=m_{2}-r_{2}-r_{3}=0$, this network has no 2-cavity.

**Summary:** Vector space $C_{1}$ has dimension $m_{1}=12$, which is the number of links in the space. There are $r_{1}=7$ linearly independent chains. Its subspace $Z_{1}=\ker(\partial_{1})$ has dimension $m_{1}-r_{1}=5$; subspace $Y_{1}=\mathrm{im}\left( \partial_{2} \right)$ has dimension $r_{2}=4$; subspace $Z_{1}$/$Y_{1}$ has dimension equal to the Betti number $\beta_{1}=m_{1}-r_{1}-r_{2}=1$. The network characteristic number is

$$\chi=m_{0}-m_{1}+m_{2}-m_{3}=8-12+5-1 \left( \mathrm{simplexes} \right)$$

$$=\beta_{0}-\beta_{1}+\beta_{2}=1-1+0 \left( Betti number \right)=0$$

Next, some other applications of the above boundary matrices, namely node-link matrices and link-face matrices, are further discussed.

A node-link matrix multiplied by its transpose gives the network degree matrix, which further yield the adjacency matrix by setting its diagonal entries to zero. For instance, from Example 1 it follows that

1 2 3 4 5 6 7 8

1 4 1 1 1 1 0 0 0

2 1 4 1 1 1 0 0 0

3 1 1 5 1 0 1 0 1

4 1 1 1 3 0 0 0 0

5 1 1 0 0 2 0 0 0

6 0 0 1 0 0 2 1 0

7 0 0 0 0 0 1 2 1

8 0 0 1 0 0 0 1 2

A node-link matrix multiplied by a link-face matrix gives the network clique matrix, which has a clique if the matrix is not zero. For instance, it follows from Ex. 1 that

(1,2,3) (1,2,4) (1,3,4) (2,3,4) (1,2,5)

1 2 2 2 0 2

2 2 2 0 2 2

3 2 0 2 2 0

4 0 2 2 2 0

5 0 0 0 0 2

6 0 0 0 0 0

7 0 0 0 0 0

8 0 0 0 0 0

A cavity matrix can be computed via $k$-clique decomposition, e.g., by multiplying the node-link matrix of the 2-clique with its transpose, one obtains

(3,6) (3,8) (6,7) (7,8)

1 0 0 0 0

2 0 0 0 0

3 1 1 0 0 0 0 1 0 0 1 0 0

4 0 0 0 0 0 0 1 0 0 0 0 1

5 0 0 0 0 0 0 0 0 0 1 1 0

6 1 0 1 0 0 0 0 0 0 0 1 1

7 0 0 1 1

8 0 1 0 1

1 0 0 0 0 0 0 0 0

2 0 0 0 0 0 0 0 0

3 0 0 2 0 0 1 0 1

4 = 0 0 0 0 0 0 0 0

5 0 0 0 0 0 0 0 0

6 0 0 1 0 0 2 1 0

7 0 0 0 0 0 1 2 1

8 0 0 1 0 0 0 1 2

The final results show that nodes 3, 6, 7, 8 have cavities (number: 2) and the cavities contain the link (number: 1) $\sigma_{36}+\sigma_{67}+\sigma_{78}+\sigma_{83}$.

**Example 2.**

The right-hand graph shown in Fig. 3 has 6 nodes and 12 links, which is a totally homogeneous network.

A basis of $C_{1}$ is

$$\left\{ \left( 1,2 \right), \left( 1,3 \right), \left( 1,4 \right), \left( 1,5 \right), \left( 2,3 \right), \left( 3,4 \right), \left( 4,5 \right), \left( 5,2 \right), \left( 2,6 \right), \left( 3,6 \right), \left( 4,6 \right), \left( 5,6 \right) \right\}.$$

Its node-link matrix $B_{1}$ is as follows, with rank $r_{1}=5$.

(1,2) (1,3) (1,4) (1,5) (2,3) (3,4) (4,5) (5,2) (2,6) (3,6) (4,6) (5,6)

**1** 1 1 1 1 0 0 0 0 0 0 0 0

**2** 1 0 0 0 1 0 0 1 1 0 0 0

**3** 0 1 0 0 1 1 0 0 0 1 0 0

**4** 0 0 1 0 0 1 1 0 0 0 1 0

**5** 0 0 0 1 0 0 1 1 0 0 0 1

**6** 0 0 0 0 0 0 0 0 1 1 1 1

In this matrix, all the links corresponding to the red “1”s, namely, $\left( 1,2 \right), \left( 1,3 \right), \left( 1,4 \right), \left( 1,5 \right), (2,6)$, constitute a spanning tree of the network, and the number of links is the number of nodes minus 1. Together with the other links corresponding to black “1”s, they form linearly independent cycles, which are cliques $\left( 1,2,3 \right), \left( 1,3,4 \right), \left( 1,4,5 \right), \left( 1,5,2 \right), \left( 2,3,6 \right), \left( 3,4,6 \right), (4,5,6)$, where the number of cycles equals the number of links minus the number of nodes and then plus 1. Usually, spanning trees are not unique; here $\left( 1,2 \right), \left( 2,3 \right), \left( 3,4 \right), \left( 2,5 \right), (2,6)$ also constitute a spanning tree. In this case, a linearly independent cycle is consisting of $\left( 1,2,3 \right), \left( 1,3,4 \right), \left( 1,5,2 \right), \left( 2,3,6 \right), \left( 3,4,6 \right), \left( 2,5,6 \right), (2,3,4,5)$.

A basis of $C_{2}$ is $\{ \left( 1,2,3 \right), \left( 1,3,4 \right), \left( 1,4,5 \right), \left( 1,5,2 \right), \left( 2,3,6 \right), \left( 3,4,6 \right), \left( 4,5,6 \right), \left( 5,2,6 \right)\}$.

Its link-face matrix $B_{2}$ is as follows, with rank $r_{2}=7$.

(1,2,3) (1,3,4) (1,4,5) (1,5,2) (2,3,6) (3,4,6) (4,5,6) (5,2,6)

(1,2) 1 0 0 1 0 0 0 0

(1,3) 1 1 0 0 0 0 0 0

(1,4) 0 1 1 0 0 0 0 0

(1,5) 0 0 1 1 0 0 0 0

(2,3) 1 0 0 0 1 0 0 0

(3,4) 0 1 0 0 0 1 0 0

(4,5) 0 0 1 0 0 0 1 0

(5,2) 0 0 0 1 0 0 0 1

(2,6) 0 0 0 0 1 0 0 1

(3,6) 0 0 0 0 1 1 0 0

(4,6) 0 0 0 0 0 1 1 0

(5,6) 0 0 0 0 0 0 1 1

Similarly, the red “1”s correspond to seven triangles consisting of a spanning tree of the network, which is a 2-chain, where the number of triangles equals the matrix rank $r_{2}=7$. Together with the rest triangle, they form linearly independent 2-cycles (a 2-cavity), where the number of cavities is $m_{2}-r_{2}=1$, namely, the whole network is a 2-cavity. This can also be verified by multiplying the face-link matrix with its transpose. All the 8 faces in sequence (number: 1) constitute 2-cavities (number: 3).

(1,2,3) 3 1 0 1 1 0 0 0

(1,3,4) 1 3 1 0 0 1 0 0

(1,4,5) 0 1 3 1 0 0 1 0

(1,5,2) 1 0 1 3 0 0 0 1

(2,3,6) 1 0 0 0 3 1 0 1

(3,4,6) 0 1 0 0 1 3 1 0

(4,5,6) 0 0 1 0 0 1 3 1

(5,2,6) 0 0 0 1 1 0 1 3

From these matrices, one can see that its Betti number is $\beta_{1}=m_{1}-r_{1}-r_{2}=0$, hence the network has no 1-cavity. Although $\sigma_{23}+\sigma_{34}+\sigma_{45}+\sigma_{52}$, $\sigma_{12}+\sigma_{26}+\sigma_{64}+\sigma_{41}$, $\sigma_{13}+\sigma_{36}+\sigma_{65}+\sigma_{51}$ all seem to be 1-cavity, they actually constitute the boundary of four triangles, which is equivalent to any of them. Furthermore, for this network, $m_{3}=0$, thus $r_{3}=0$, since their Betti number is $\beta_{2}=m_{2}-r_{2}-r_{3}=1$, there is only one 2-cavity. The 2-cavity is a closed structure enclosed by triangles. The network characteristic number is $=6-12+8 \left( \mathrm{simplexes} \right)=1-0+1 \left( Betti numbers \right)$ $=2$.

This example shows that, although the total number of linearly independent cycles so obtained is always the same, which is equal to the number of links minus the number of nodes and then plus 1, the resulting cycles might be different, and sometimes false cavities like $(2,3,4,5)$ might be generated. To avoid such false cavities, computing the matrix rank is necessary. Besides, the above two examples both show that, due to linear dependence, not all the smallest cycles (triangles) are included in the linearly independent cycles. Although this issue might be resolved by using the smallest cycle algorithm ^10^, the best approach is to study and apply the simplex algorithm, which can compute the most important invariants for complex networks—characteristic number and Betti number.

The network shown in Figure 3 is merged by the two networks shown in Figure 6 through two connections.

**Section 2**: Simulation results.

Network 1 Network 2 Network 3 Network 4

Number of recovered nodes Number of recovered nodes Number of recovered nodes Number of recovered nodes

3.140000 3.570000 2.230000 4.710000

3.070000 3.360000 2.220000 4.610000

3.020000 3.020000 2.140000 4.580000

2.970000 2.950000 2.020000 4.530000

2.900000 2.840000 1.810000 4.450000

2.890000 2.810000 1.770000 4.390000

2.730000 2.680000 1.690000 4.360000

2.710000 2.620000 1.680000 4.310000

2.690000 2.590000 1.580000 4.240000

2.650000 2.550000 1.540000 4.120000

2.630000 2.500000 1.500000 4.090000

2.590000 2.450000 1.410000 4.030000

2.560000 2.380000 1.410000 3.960000

2.560000 2.290000 1.350000 3.920000

2.550000 2.290000 0.840000 3.880000

2.500000 2.130000 0.650000 3.730000

2.440000 2.080000 0.330000 3.650000

2.320000 2.080000 0.290000 3.650000

2.160000 1.130000 0.260000 3.580000

2.040000 0.490000 0.260000 3.420000

The above is the result for each node, and the average results of 20 nodes are:

2.656000 2.440500 1.349000 4.110500
